# The Na^+^/K^+^-ATPase: A potential therapeutic target in cardiometabolic diseases

**DOI:** 10.3389/fendo.2023.1150171

**Published:** 2023-02-28

**Authors:** Milan Obradovic, Emina Sudar-Milovanovic, Zoran Gluvic, Katarina Banjac, Manfredi Rizzo, Esma R. Isenovic

**Affiliations:** ^1^ Department of Radiobiology and Molecular Genetics, “VINČA“ Institute of Nuclear Sciences - National Institute of thе Republic of Serbia, University of Belgrade, Belgrade, Serbia; ^2^ University Clinical-Hospital Centre Zemun-Belgrade, Clinic of Internal medicine, School of Medicine, University of Belgrade, Belgrade, Serbia; ^3^ School of Medicine, Promise Department, University of Palermo, Palermo, Italy

**Keywords:** Na^+^/K^+^-ATPase, cardiometabolic diseases, cardiovascular diseases, type 2 diabetes mellitus, therapy

## Abstract

Cardiometabolic diseases (CMD) are a direct consequence of modern living and contribute to the development of multisystem diseases such as cardiovascular diseases and diabetes mellitus (DM). CMD has reached epidemic proportions worldwide. A sodium pump (Na^+^/K^+^-ATPase) is found in most eukaryotic cells’ membrane and controls many essential cellular functions directly or indirectly. This ion transporter and its isoforms are important in the pathogenesis of some pathological processes, including CMD. The structure and function of Na^+^/K^+^-ATPase, its expression and distribution in tissues, and its interactions with known ligands such as cardiotonic steroids and other suspected endogenous regulators are discussed in this review. In addition, we reviewed recent literature data related to the involvement of Na^+^/K^+^-ATPase activity dysfunction in CMD, focusing on the Na^+^/K^+^-ATPase as a potential therapeutic target in CMD.

## 1 Introduction

Cardiometabolic diseases (CMD) are a direct consequence of the modern lifestyle and represent a step forward in the development of multisystem diseases such as cardiovascular diseases (CVD) and diabetes mellitus (DM) (1, 2). The prevalence of CMD achieves epidemic proportion, estimated at approximately 25% at the global level (3, 4). An unhealthy diet combined with sedentary behaviour, smoking, alcohol use and socioeconomic aspects is a substantial risk factor for the development of cluster metabolic disorders, including obesity, hypertension, dyslipidaemia and impaired glucose regulation (5, 6). Aside from prevention, there are numerous therapeutics for CMD treatment on the market, most of which are designed to improve insulin action and lipid-lowering. However, the dramatic increase in the prevalence of CMD and the inadequacy of current therapy point to the need for new therapeutic targets.

The sodium/potassium adenosine-triphosphatase (Na^+^/K^+^-ATPase) is an essential plasma membrane enzyme that maintains ion homeostasis, cell volume and contractility, electrical signaling, membrane trafficking and vascular tone (7). The Na^+^/K^+^-ATPase is the target of several controlling mechanisms. Hormones up-regulate and downregulate Na^+^/K^+^-ATPase activity/expression, which primarily comes to the fore in different CMD (8–12). Also, Na^+^/K^+^-ATPase functions as a receptor for cardiotonic steroids (CTS), with downstream molecular response affected by CTS concentration. Higher concentrations of CTS (mM range) lead to reverse the inhibition of Na^+^/K^+^-ATPase activity, causing a transient cytotoxic effect and, most importantly positive inotropic effect (13). Precisely for this reason, cardiac glycosides have been used for a long time as a drug to strengthen the force of the heartbeat in numerous heart disorders (14). In addition, CTS were among the 200 most frequently prescribed drugs in 2018 year in the USA (15). Particular mechanisms of Na^+^/K^+^-ATPase regulation arise after CTS binding to the specific site at α subunit of Na^+^/K^+^-ATPase, but at low CTS concentrations (≤ nM) which is insufficient for ion transport inhibition (16). Cell signaling, intracellular Ca^2+^ oscillations, gene transcription, growth, and proliferation are all activated as a result (17, 18). Since its discovery, Na^+^/K^+^-ATPase has been the subject of numerous studies, but the regulation mechanism remains unknown.

Given that CMD alters Na^+^/K^+^-ATPase activity and/or subunit expression (8, 9, 13, 19), it represents a promising therapeutic target (20, 21). Furthermore, basic and clinical studies show that improving Na^+^/K^+^-ATPase function is directly related to improving various pathological conditions of the cardiovascular system (22). In this review, we discussed recent literature data on Na^+^/K^+^-ATPase regulation in CMD as a potential target for new approaches to treating these pathologies.

## 2 Na^+^/K^+^-ATPase structure

**Figure 1:**
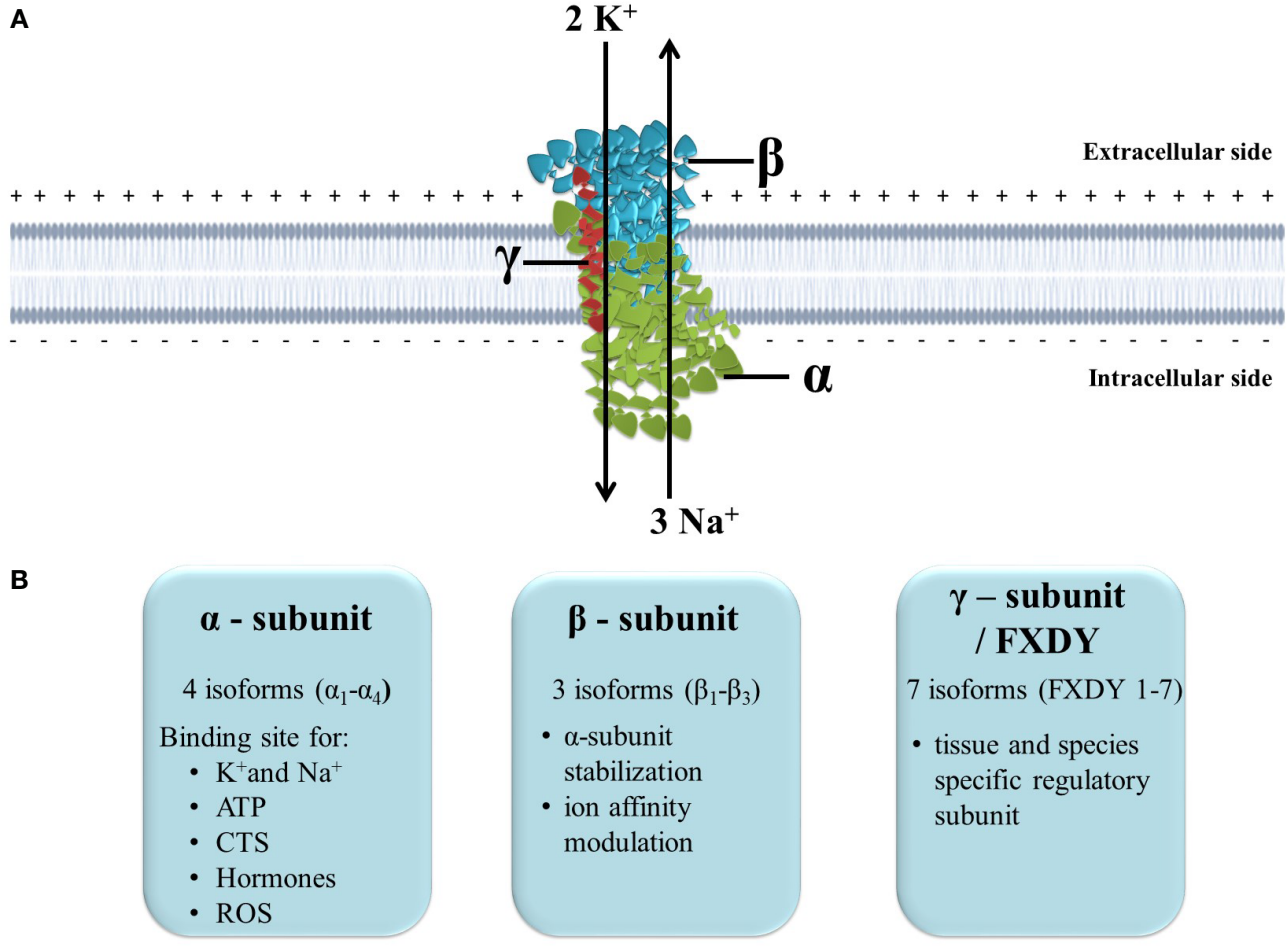
The structure and function of Na^+^/K^+^-ATPase. **(A)** Position of Na^+^/K^+^-ATPase in the plasma membrane and maintenance of ion homeostasis; **(B)** Subunits isoforms and specific function. ATP, adenosine triphosphate; CTS, cardiotonic steroids; ROS, reactive oxygen species.

The transmembrane protein, Na^+^/K^+^-ATPase transports K^+^ ions into the cell and Na^+^ ions out of the cell, and since the process requires transporting ions against their concentration gradients, Na^+^/K^+^-ATPase uses the energy derived from hydrolysis of ATP. It is composed of a ∼100 kDa catalytic α subunit, a heavily glycosylated ∼45 kDa β subunit, and a regulatory subunit, often referred to as γ-subunit (∼10 kDa), that belongs to an FXYD group of proteins (**Figure 1**) (13, 23). The subunits display multiple isoforms, four α subunit isoforms and three β subunit isoforms, which can assemble in 12 different Na^+^/K^+^-ATPase isozymes with tissue-specific different functional activities. Seven tissue and Na^+^/K^+^-ATPase isozymes are also specific γ-subunit isoforms (23). α-subunit has a large intracellular domain with ATP-binding and phosphorylation site, a transmembrane domain composed of ten segments responsible for ion transport, and an extracellular domain with binding sites for cardiac steroids (24). β-subunit has an essential role in the α-subunit assembly, and additionally, it increases α-subunit stability and modulates its affinity of ions (24). On the other hand, γ-subunits are tissue-specific and act as Na^+^/K^+^-ATPase modulatory proteins. Whereas heart tissue specific γ-subunit is phosholemman (PLM), which disinhibits Na^+^/K^+^-ATPase in its phosphorylated form, increasing Na^+^ efflux (25). The γ-subunit influences the affinity of the Na^+^/K^+^-ATPase for ions and ATP, in addition to the transport and stabilization properties (26).

Normal functioning Na^+^/K^+^-ATPase in the plasma membrane is vital for mammalian cells since it maintains Na^+^ and K^+^ electrochemical gradients across the plasma membrane. Many ion transporters and channels utilize these chemical gradients to transport ions, minerals, sugar and amino acids (23). Therefore it is involved in ion homeostasis regulation, intracellular pH regulation, Ca^2+^ signaling, fluid and volume homeostasis regulation and renal salt reabsorption (13). Additionally, the ion gradient Na^+^/K^+^-ATPase creates across the plasma membrane is essential for generating action potential that sustains cardiac muscle contraction and neuronal communication. Finally, novel studies confirm that Na^+^/K^+^-ATPase also acts as a signal transducer since it is a cardiotonic steroids receptor and can activate intracellular protein kinases (23, 27).

### 2.1 The molecular mechanism of Na^+^/K^+^-ATPase regulation

In the cardiovascular system, Na^+^/K^+^-ATPase is important in regulating vascular tone and cardiac remodelling (28). Animal hearts express the α_1_ isoform dominantly or in combination with the α_2_ and/or α_3_ isoform. Considering rodent adult cardiomyocytes, they mainly express the α_1_ isoform and α_2_ isoform (<25%) (29), while human adult cardiomyocytes have all three isoforms expressed (13). Since α_1_ and α_2_ isoforms are present in different ratios and with differential distribution in cardiac cells, it has been suggested that they have different functions. Both α_1_ and α_2_ isoforms in the heart have physical and functional associations with Na^+^/Ca^2+^ exchangers, thus favouring Ca^2+^ influx rather than Ca^2+^ efflux, which leads to increased contractility (29–31). Moreover, the α_2_ isoform is approximately five times more present in the T-tubules, where the Na^+^/Ca^2+^ exchanger is located (32). At the same time, data indicate that the α_1_ isoform in the heart regulates cell growth and survival *via* maintaining a global pool of Na^+^ throughout the cell. On the other hand, the α_2_ isoform regulates Ca^2+^ concentration in cells *via* regulating local Na^+^ and Ca^2+^ concentrations in sarcolemma/sarcoplasmic reticulum microdomains, thereby regulating contractility and hypertrophy (29–31). Furthermore, α_2_ isoform overexpression has a protective effect from pressure overload caused by cardiac dysfunction; thus, this isoform probably regulates cardiac pathological hypertrophy (29, 30). Numerous studies have also implicated aberrant Na^+^/K^+^-ATPase and PLM expression, reduction in Na^+^/K^+^-ATPase activity and subsequent increase in intracellular Na^+^ and Ca^2+^ concentrations in diseased heart (31, 33). Chronic increase in intracellular Na^+^ and Ca^2+^ concentrations lead to maladaptive cardiac hypertrophy and arrhytmogenesis (31). Additionally, several pathophysiological conditions such as insulin resistance (IR), obesity and hypertension are associated with defects in normal Na^+^/K^+^-ATPase function (8, 9, 13, 19).

**Figure 2:**
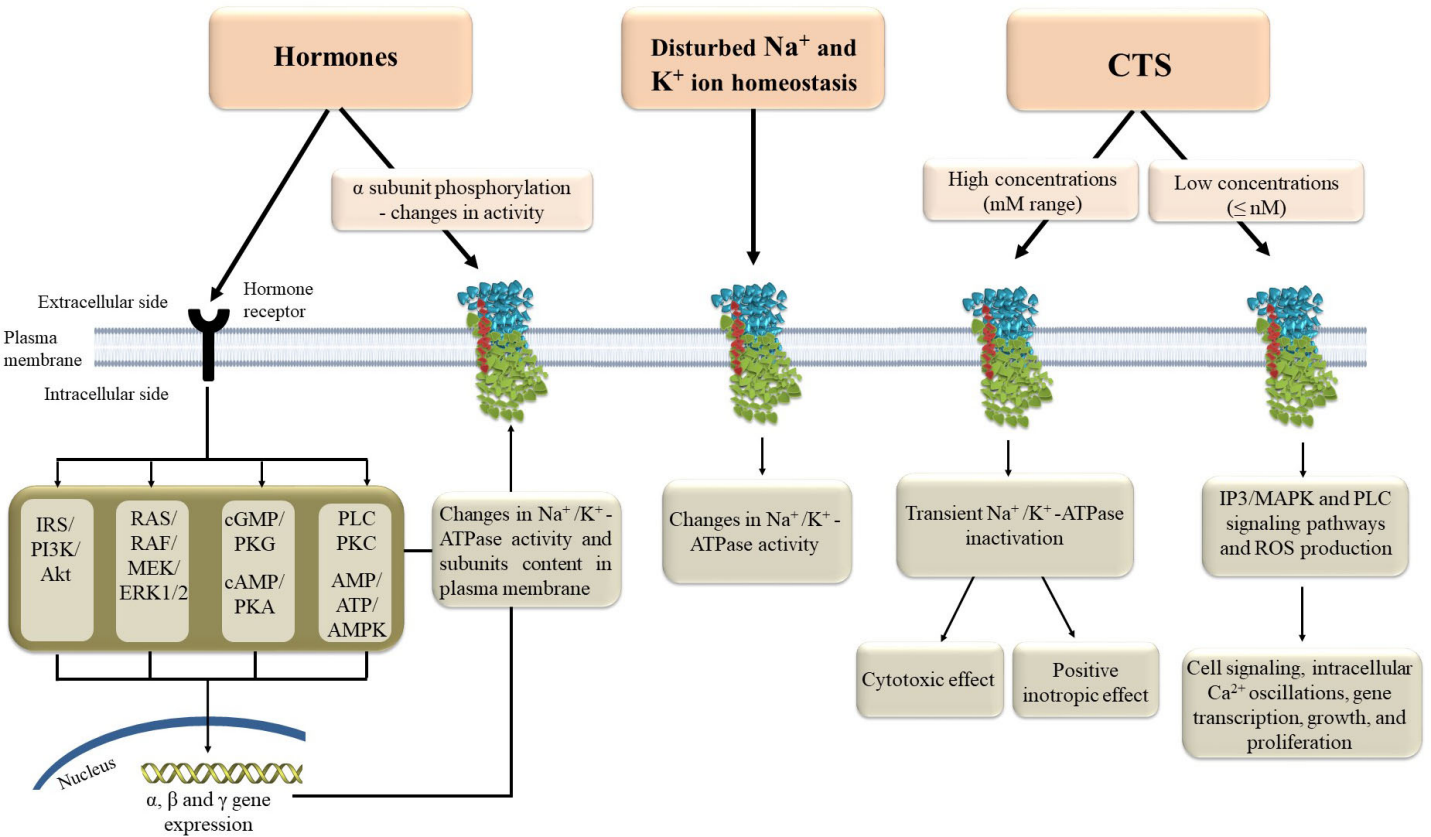
Molecular mechanisms of Na^+^/K^+^-ATPase regulation. AMP - adenosine monophosphate, AMPK – AMP-activated protein kinase, ATP - adenosine triphosphate, CTS – cardiotonic steroids, ERK1/2 – extracellular signal-regulated kinases 1 and 2, IP3 – inositol triphosphate, MAPK – mitogen-activated protein kinase, PKA, protein kinase A; PKC, protein kinase C; PKG, cGMP-activated protein kinase; PLC, phospholipase C; ROS, reactive oxygen species.

Na^+^/K^+^-ATPase regulation is a crucial and highly complex process on various levels (**Figure 2**). Concerning tissue-specific mechanisms of Na^+^/K^+^-ATPase regulation, there are local and systemic regulatory mechanisms. Intracellular and extracellular Na^+^ and K^+^ concentrations are the most important local regulatory mechanism, along with hypoxia, purines, oxidative stress, pH, nitric oxide and ATP, that influence activity of Na^+^/K^+^-ATPase (34, 35). On the other hand, hormones are major factors in the systemic regulation of Na^+^/K^+^-ATPase. Hormones regulate Na^+^/K^+^-ATPase cell surface expression and activity, provoking protein kinase phosphorylation (35–41). Nonetheless, translocation from intracellular compartments to the plasma membrane is controlled by α-subunit phosphorylation, a type of posttranslational modification (12, 42, 43). Furthermore, the α subunit contains several serine, threonine, and tyrosine residues that can be phosphorylated by various kinases, influencing Na^+^/K^+^-ATPase activity (44). Besides phosphorylation, Na^+^/K^+^-ATPase can be modified *via* glutathionylation, which causes its inactivation (35). Additionally, except for regulating Na^+^/K^+^-ATPase cell surface expression, hormones can up-regulate α and β gene transcription, which determines the total cell content of Na^+^/K^+^-ATPase subunits along with the degradation rate [9]. Insulin, as one of the most potent regulators of Na^+^/K^+^-ATPase, increased its activity and translocation of subunits to the cell membrane *via* phosphatidylinositol 3-kinase (PI3K), protein kinase C (PKC), and extracellular signal-regulated kinases 1 and 2 (ERK1/2) (42, 43, 45). In contrast, leptin decreased Na^+^/K^+^ATPase activity in the rat kidney *via* the PI3K pathway (46). Angiotensin II (Ang II), insulin-like growth factor 1 (IGF-1) and estradiol stimulate Na^+^/K^+^-ATPase activity and gene expression in primary cultured rat vascular smooth muscle cells *via* PI3K, protein kinase B (Akt), and ERK1/2 (9, 37, 38). Estradiol also increased Na^+^/K^+^-ATPase activity and expression in the heart of rats *via* signaling pathways that involve stimulation of insulin receptor substrate 1 (IRS-1)/PI3K/Akt/ERK1/2 and suppression of Ang II receptor type 1, Rho A, and Rho-associated kinase cascade (8, 47). Furthermore, Ang II inhibits IGF-1-stimulated Na^+^/K^+^-ATPase activity in VSMC *via* PI3K/Akt signaling (37), whereas IGF-1 overexpression reduced Ang II production and oxidative stress in mouse cardiomyocytes (48). Several studies have shown that the signaling pathways that regulate IGF-1 and estradiol are crosslinked, implying that these hormones may have a combined effect on the regulation of Na^+^/K^+^-ATPase (49). This interactive effect of hormones indicates a complex mechanism of Na^+^/K^+^-ATPase regulation *in vivo* where tonic hormone release simultaneously influences Na^+^/K^+^-ATPase and balances its activity.

New researches also confirm that γ-subunits or FXYD proteins can modulate Na^+^/K^+^-ATPase binding and function *via* protein-protein interactions and consequent post-translational modifications (50). These effects, along with the FXYD proteins expression, are also regulated by hormones (35). Since FXYD proteins are tissue-specific, post-translational modifications fine-tune Na^+^/K^+^-ATPase binding and function according to tissue-specific needs (32). In addition, FXYD proteins can also be substrates for post-translational modulation, which modifies their regulatory function (32). Finally, cardiac steroids bind to the extracellular domain of α- subunit and stabilize and modify Na^+^/K^+^-ATPase to support its different functions. Therefore, cardiac steroids can have a positive inotropic effect through Na^+^/K^+^-ATPase inhibition and the effect of activation of different signal transduction pathways mediated by Na^+^/K^+^-ATPase. In comparison, higher cardiac steroids concentrations induce Na^+^/K^+^-ATPase inhibition, while sub-inhibitory concentrations induce activation of mitogen-activated protein kinase signal cascades, mitochondrial reactive oxygen species (ROS) production, and the phospholipase C signaling pathway (23). In addition, cardiac steroids can modulate Na^+^/K^+^-ATPase sensitivity to different regulatory proteins (51).

## 3 CMD

The emergence of CMD risk factors is unpredictable and dynamic. Cardiovascular and metabolic disruptions most often cause CMD, and a long-lasting CMD, including DM and different cardiovascular pathologies, are the main causes of death worldwide. The incidence and prevalence of CMD have increased in parallel with the rise in obesity, DM and hypertension (52). Since mortality has reduced during the past few years in high-income nations but increased in low- and middle-income countries, increases in the prevalence of CMD, such as hypertension, obesity, dyslipidaemia and DM, and their major risk factors have not been uniform (53). Cardiometabolic disorders can occur substantially before the clinical appearance of diseases. CMD-related complications are complex and multifactorial disorders, but in most cases, preventable. Many factors, such as changes in living environments, unhealthy diets, specific lifestyles, physical inactivity, and genetic and epigenetic factors, may be involved in CMD development (52). Early and accurate predictors of CMD are of great importance since the delay or prevention of morbidity is achievable *via* pharmacological treatments and lifestyle modulation (54–56). Timely treatment of these detrimental factors is important in their progressive and ultimate transformation into more complicated CMD. Novel mechanisms implicated in the development of CMD may open up new prognostic and therapeutic avenues.

Over the last decade, it has been generally recognized that genetic mutations are engaged in different CMD (52), including hypertension (57), impaired lipid metabolism and lipotoxicity (58). In addition to defective genes, frequent inflammation is also one of the pathological driving forces involved in various CMD (59). Pathophysiological factors, such as proinflammatory cytokines: resistin, interleukin (IL)-6, tumour necrosis, factor-alpha (TNF-*α*), and IL1*β*, as well as interactions among them and also with the molecules of the insulin signaling cascade, are involved in IR occurrence (52, 59). In addition to dysfunctional insulin signaling, proinflammatory cytokines are implicated in impaired endothelial function and dyslipidemia (52), both involved in CMD.

Many cardiometabolic complications, including obesity, DMT2, hyperlipidemia, dyslipidemia, nephropathy, hypertension, and nonalcoholic fatty liver disease, are closely interrelated (52). Among the major causes of CMD and related complications is DM. Obesity usually predisposes to DM, especially central obesity per se, and is associated with severe comorbidities, influencing every system of organs, particularly affecting cardiometabolic comorbidities (60). Moreover, obesity is associated with an increased risk for CVD independently from other CVD risk factors and is also considered a modulator of other CVD risk factors. Thus, treating obesity should be the most important management strategy to reduce cardiometabolic risk (60, 61). Furthermore, it is considered a chronic metabolic disorder associated with chronic low-grade inflammation and results in marked alterations of proinflammatory cytokines, adipokines, and other molecules affecting CVS function and CMD development. In an observational cohort study in which 1.3 million overweight or obese adults participated, four commonly observed cardiac risk factors were found: the prevalence of hypertension, prediabetes, decreased HDL and elevated TG. An earlier study by National Health and Nutrition Examination Survey (NHANES), which included individuals with diabetes, showed that 52% of adults overweight and 32% of adults with obesity had no cardiac risk factors or only one, suggesting that different phenotypes of obesity, such as subcutaneous versus abdominal fat, may pose various health risks (62, 63). The authors concluded that being overweight or obese increases cardiometabolic risk, but the quantity and developed cardiac risk factors differed substantially by age, even among participants with morbid obesity (62, 63).

Furthermore, vitamin D effects on insulin sensitivity may be compromised in obese individuals (64), and in these individuals, hyperinsulinemia and/or IR may be responsible for reduced vitamin D concentration, which underscores this paradigm (52). In addition, alterations at a hormonal, inflammatory and endothelial level associated with obesity induce stimulation of several factors contributing to the hypertensive state and development of CVD and cardiovascular morbidity. The most recognized factors connecting obesity and hypertension are impaired sodium homeostasis, endocrine alterations, altered hemodynamics, autonomic nervous system imbalance, renal dysfunction, oxidative stress and inflammation, and vascular injury (65).

The development of complications and increased mortality influenced by obesity indirectly affect other risk factors such as IR, dyslipidemia, and hypertension (66). In addition, an important link between obesity and CVD development is dyslipidemia (67). Dyslipidemia occurs when the levels of triglyceride (TG), small dense LDL (sdLDL) particles, very low-density lipoprotein (VLDL) cholesterol and total cholesterol are increased, while high-density lipoprotein (HDL) cholesterol levels are decreased (68, 69). Persons with visceral adiposity usually have indicators for CVD development, such as an increased ratio of apolipoprotein (Apo) B to Apo A1 (70), a rise in sdLDL particles (71), and low HDL cholesterol level (72). In the last decade, dyslipidemia occurring due to IR and obesity has been recognized as “metabolic dyslipidemia” (73). Its main features are increased levels of TG accompanied by decreased HDL cholesterol level, while LDL cholesterol level could be mildly increased or optimal, even though the number of LDL particles (LDL-P) can also be elevated. Also, atherogenic lipoproteins, such as lipoprotein(a) (Lp (a)), are critical in the development of various CVD (74), leading to CMD (52). In addition, endothelial and vascular dysfunction caused by obesity leads to CVD (75). Furthermore, obesity predisposes to heart disease through various mechanisms, including causing structural and functional changes in the heart, affecting heart morphology and leading to pathological heart hypertrophy, characterized by cardiomyocyte enhancement and increased protein synthesis (76, 77). However, it is not accompanied by a rise in capillaries supplying the myocardium, finally leading to ischemic changes in the myocardium (78).

Among others, in patients with CMD, response to ischemic insults may also be impaired. Patients with cardiovascular risk, especially patients with hypertension and diabetes, exhibited an abnormal reactive hyperemic response to ischemic insults, which are associated with myocardial infarction (52, 59).

Many difficult problems must be solved to improve CMD diagnosis, prognosis, therapy, and management. Cardiometabolic risks are a complex group of disease entities, and risk assessment, prediction, and management are also difficult because the underlying causes that promote or precipitate cardiac risk factors in these metabolic diseases are unknown.

## 4 Na^+^/K^+^-ATPase and CMD

**Figure 3:**
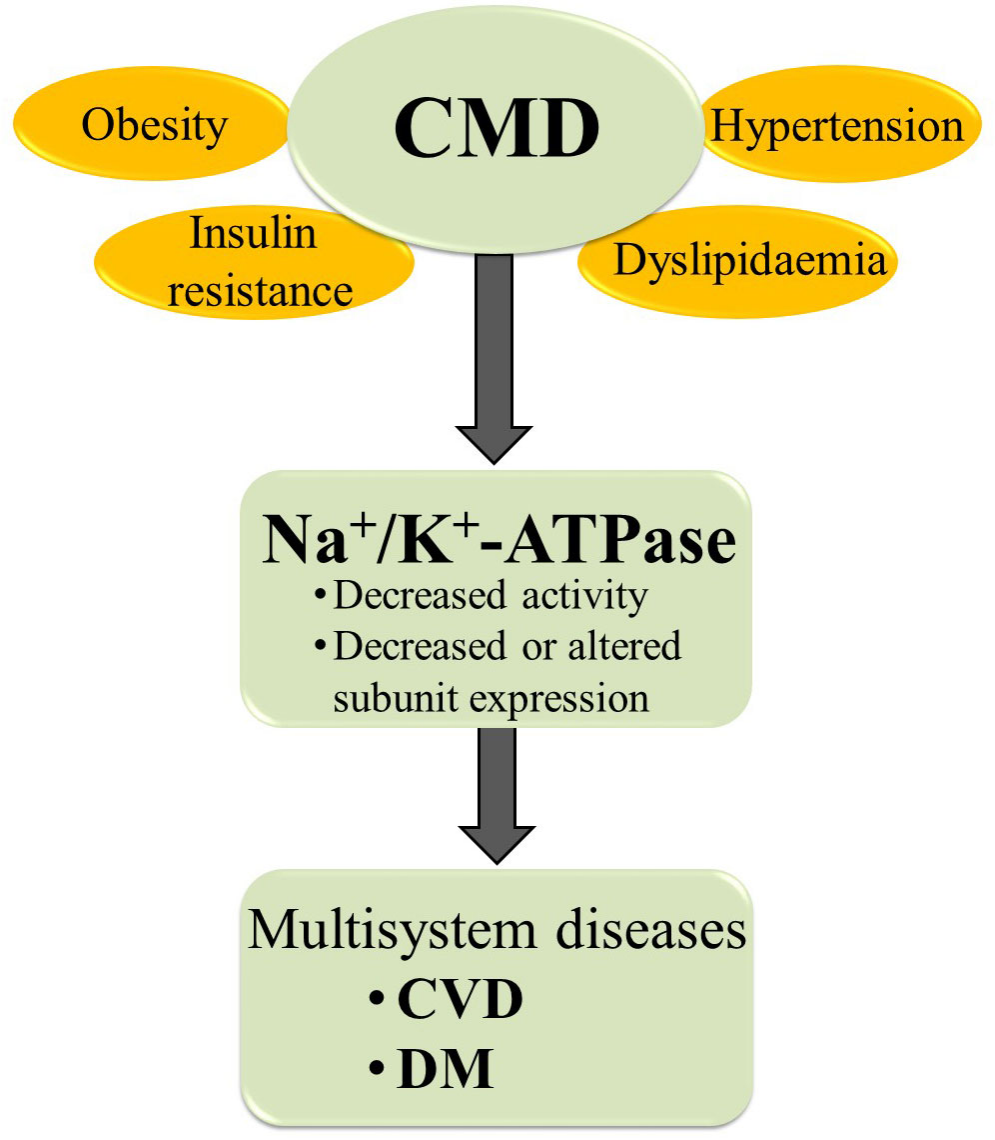
The relationships between CMD and Na^+^/K^+^-ATPase. CMD, cardiometabolic diseases; CVD, cardiovascular diseases; DM, diabetes mellitus.

Altered Na^+^/K^+^-ATPase activity/expression is the basis for vascular complication and cardiac dysfunction in different CMD (**Figure 3**) (8, 9, 19, 79–82). Decreased Na^+^/K^+^-ATPase activity and high concentrations of Na^+^ in cytosol lead to impaired myocardial contractility in advanced heart failure (83). The link between CMD and altered Na^+^/K^+^-ATPase activity is somewhat predictable given that CMD causes changes in hormone levels, most notably insulin, insulin-like growth factor 1, angiotensin II (Ang II), estradiol, and leptin, all of which are potent regulators of the Na^+^/K^+^-ATPase (12, 49). The function of Na^+^/K^+^-ATPase is impaired at different levels of regulation in hearts, aorta and erythrocytes in human and animal models of induced obesity, insulin resistance and hypertension (**Table 1**) (8, 19, 84–92, 94–101, 104–107). It has been demonstrated that leptin reduces Na^+^/K^+^-ATPase activity in fibroblasts (108), which may be important in the obese state frequently associated with hyperleptinemia. Evidence suggests that long-term activation of Na^+^/K^+^-ATPase signaling may promote cardiac fibrosis and the development of heart dysfunction (109–113). Furthermore, using Na^+^/K^+^-ATPase signaling antagonists, such as pNaKtide, has shown promise in reducing organ fibrosis (109, 113). We also found that a high-fat diet induces obesity and IR in rats, resulting in decreased activity and α_1_ and α_2_ subunits of expression of Na^+^/K^+^-ATPase in cardiac tissue, which is accompanied by heart hypertrophy (8, 76). In addition, decreased Na^+^/K^+^-ATPase activity is detected in erythrocytes of obese, IR and DM patients (19, 102, 103, 114). Increased Ang II and (ROS) inhibit Na^+^/K^+^-ATPase by glutathionylation of β_1_ subunit that may have pathophysiological effects in the cardiovascular system of obese and DM patients (115–117). The activity of cardiac Na^+^/K^+^-ATPase is decreased in hypertensive male rats (91). Also, earlier studies reported altered expression of α_1_ and α_2_ subunits of Na^+^/K^+^-ATPase in the aorta and heart of hypertensive rats (118, 119). Mice with an ouabain-resistant α_2_ subunit of Na^+^/K^+^-ATPase are protected from hypertension development after treatment with adrenocorticotropic hormone (120, 121). Genetic silencing of the α_2_ subunit of the Na^+^/K^+^-ATPase decreased pathological heart hypertrophy and cardiac remodeling (93, 122). The Na^+^/K^+^-ATPase signaling is activated with ROS and CTS (33, 123). However, in pathophysiological conditions such as obesity and related disorders, increased ROS and CTS promote Na^+^/K^+^-ATPase signaling, leading to the overproduction of ROS and inflammatory markers creating an oxidant amplification loop that consequently alters the metabolic profile (124). Recent research reveals an important role of Na^+^/K^+^-ATPase in autosis, that is characterized as an autophagy-dependent non-apoptotic form of cell death in different (125, 126). The increased interaction of Na^+^/K^+^-ATPase with the autophagy protein Beclin 1 was detected in ischemic conditions of hearts (127). Further studies are needed to enhance our knowledge of Na^+^/K^+^-ATPase in oxidant amplification and autosis, which may be a target option for CMD treatment.

**Table 1:** The link between cardiometabolic diseases and Na^+^/K^+^-ATPase activity/expression.

Cardiometabolic diseases	Study group	Effect on Na^+^/K^+^ ATPase	Organ/tissue	Ref.
Obesity	Rat	↓expression and activity	Heart	(8)
	Human	↓activity	Erythrocytes	(84)
	Mice and human	↓activity	Liver and kidney	(19)
	Rat	↑cortical α1 subunit abundance	Kidney	(85)
	Rat	↓α_1_ subunit expression and ↑α_1_ subunit content in plasma membrane	Heart	(86)
Hypertension	Rat	↓affinity for Na^+^	Heart	(87)
	Mice	↑α_2_ subunit expression	Aorta	(88)
	Rat	↓expression and activity	Kidney	(89)
	Rat	↑activity	Kidney	(90)
	Rat	↓affinity for Na^+^	Heart	(91)
	Human	↓activity	Erythrocytes	(92)
	Mice	↑α_2_ subunit expression	Heart	(93)
Insulin resistance and diabetes	Rat	↓expression and activity	Heart	(94)
	Rat	↓expression and activity	Heart	(95, 96)
	Rat	↓α_1_ subunit expression ↑α_1_ subunit content in the plasma membrane	Heart	(86)
	Rat	↑ α_1_ subunit content and ↓ Na^+^/K^+^ ATPase activity in the plasma membrane	Skeletal muscle	(97)
	Rat	↓α_1_ and β_1_ subunit expression and ↓Na^+^/K^+^ ATPase activity	Kidney	(98)
	Rat	↓ activity	Heart	(99)
	Rat	↓expression and activity	Heart	(100)
	Rat	↓expression and activity	Aorta	(101)
	Human	↓ activity	Erythrocytes	(102)
	Human	↓ activity	Erythrocytes	(103)

↑ - increase; ↓ - decrease.

## 5 Therapeutic target

Because of its critical role in numerous cellular processes that extrapolate to overall body function, the Na^+^/K^+^-ATPase is a promising drug target. Even though Na^+^/K^+^-ATPase was studied decades ago, the mechanism of Na^+^/K^+^-ATPase regulation is very complex and is still not fully understood. An important issue in Na^+^/K^+^-ATPase regulation is balancing its activity and its function as a receptor through which signaling pathways are activated.

Since the discovery of Na^+^-K^+^-ATPase, it has been evident that modulation of its activity could serve as a pharmacology and therapeutic target (128). According to this, Na^+^/K^+^-ATPase activity inhibition can be promoted by various agents (i.e. endogenous and exogenous cardiac steroids), but it can also be seen in different pathologies, such as heart failure, with a significant decrease (40%) in myocardial Na^+^/K^+^-ATPase concentration (14, 129–131). Insulin and β adrenergic agonists are important in increasing the amount of Na^+^/K^+^-ATPase, which promotes K^+^ transport into cells (131, 132). Because Na^+^/K^+^-ATPase is a non-specific receptor for ROS, the Na^+^/K^+^-ATPase-Src oxidant amplification loop is important in the ageing process, obesity, and atherosclerotic CVD (123, 133).

The pharmacological mechanism of CTS cardiovascular effects is based on the inhibition of Na^+^/K^+^-ATPase, followed by an increase in intracellular Ca^2+^ concentration, and then the promotion of positive inotropic and negative chronotropic effects (134, 135). CTS are classified as endogenous or exogenous (cardiac glycoside) based on their source. Endogenous CTS functions in mammals as endogenous digitalis-like factors (135). Among the most extensively studied endogenous CTS are cardenolides (ouabain and digoxin) and bufadienolides (marinobufagenin, telocinobufagin, and 19-Norbufalin) (14, 136). Marinobufagenin and its reduced form, telocinobufagin, were found in the bodily fluids of patients suffering from myocardial infarction, acute renal failure, end-stage renal disease, and heart failure (137–141). Patients with hypertension and pregnant women with preeclampsia had higher ouabain levels (142, 143). *Digitalis lanata* and *Digitalis purpurea* are the primary sources of cardiac glycosides (134). Digitoxin, digoxin, lantoside C, and strophanthin K are clinical preparations in use (135).

A disturbed transarcolemmal Na^+^ gradient characterizes ventricular wall hypertrophy and dilation (144, 145). Increased intracellular Na^+^ content inhibits Ca^2+^ mitochondrial uniporter/exchanger function, causing the mitochondria to become metabolically exhausted due to an ATP supply-demand mismatch (146). Furthermore, mitochondrial dysfunction promotes the production of ROS (147). Ouabain, a cardiotonic glycoside, binds to the subunit and inhibits Na^+^/K^+^-ATPase (148). Digoxin and digitoxin inhibit the Na^+^/K^+^-ATPase directly (149). Such Na^+^/K^+^-ATPase inhibition in the myocardium causes an increase in K^+^ efflux at the same time as intracellular Na^+^ accumulation, resulting in decreased Na^+^/Ca^2+^ channel exchanger activity and an increase in the sarcoplasmic reticulum and cytosolic Ca^2+^ content in cardiomyocytes (150). Furthermore, digitalis glycosides attenuate Ca^2+^ influx in cells (13). The net effect is increased intracellular Ca^2+,^ which strengthen heart contractility (129). Furthermore, cardiac glycosides favour a longer atrioventricular node refractory period and sinoatrial depression (both beneficial in atrial fibrillation), an increase in cardiomyocyte automatism (which promotes ventricular arrhythmogenic foci), and a decrease in atrioventricular impulse conduction (151). Digitalis additionally slows heart rate through vagal activation (152). Digoxin is now used to treat persistent heart failure symptoms in patients already receiving modern therapy and control heart rate in patients with atrial fibrillation and heart failure, but it does not affect mortality rates (151–153). Other pharmacological agents, in addition to cardiotonic glycosides, influence Na^+^/K^+^-ATPase activity. Diuretic-induced K^+^ loss and secondary hyperaldosteronism associated with heart failure reduce myocardial Na^+^/K^+^-ATPase activity (154), whereas angiotensin-converting enzyme inhibitors (ACEi) and spironolactone may stimulate myocardial Na^+^/K^+^-ATPase activity (130, 155). Aside from plant-derived cardiotonic glycosides, endogenous vertebrate-derived aglycones such as bufalin and marinobufagenin, whose production in the adrenals and possibly hypothalamus is primarily under humoral control (ACTH, Ang II) are also detected as Na^+^/K^+^-ATPase inhibitors (156–158). Subnanomolar concentrations of plant- and vertebrate-derived glycosides have been found in various diseases such as hypertension (142), renal failure (159), and atherosclerotic CVD (160, 161). In addition to the beneficial roles of endogenous cardiotonic steroids in heart contractility, heart rate control, natriuresis, and blood pressure regulation, chronic exposure causes deleterious effects such as ventricular and vascular wall remodelling, myocardial fibrosis, and arrhythmia risks (14). Exogenous CTS (digoxin, digitoxin) is intended for patients with heart failure with reduced ejection fraction and AF with rapid ventricular rate, especially if previously approved therapy (diuretics, angiotensin-converting enzyme inhibitors (ACEI)/angiotensin receptor blockers, -blockers, and aldosterone receptor antagonists) fails (162–167). Digoxin improves cardiac function and prognosis, and lowers hospitalization rates in HF patients but has no effect on all-cause mortality (168, 169). In contrast, the cardiovascular remodeling caused by long-term CTS exposure promotes the development of cardiac fibrosis pro-arrhythmic foci (170). Because of digoxin’s narrow therapeutic range should be used with caution in elderly, malnourished, and hypokalemic patients (167). The possibility of a deleterious effect of concomitantly administered digoxin, the most common type of CTS in clinical use, arises in patients with already elevated levels of endogenous CTS (14, 135). Digitoxin could be used in patients with impaired renal function (167, 171).

Reduced Na^+^/K^+^-ATPase activity and expression are detected in chronic kidney-related heart injury (172). Zheng et al. show that targeting the DR extracellular region (897DVEDSYGQQWTYEQR911) of α_1_ subunit’s Na^+^/K^+^-ATPase with DRm217 antibody stimulates Na^+^/K^+^-ATPase activities and protects ischemic injury and cardiac remodeling injury in rats (20). β3 adrenoceptor agonist increased Na^+^/K^+^-ATPase activity and reduced indices of organ congestion in a rabbit model, suggesting that decreased Na^+^/K^+^-ATPase activity may serve as a treatment target in a state of congestive heart failure (21).

The modulation of myocardial Na^+^/K^+^-ATPase activity and expression by different exogenous and endogenous cardiac steroids in animal models helps unravel all the molecular mechanisms in which Na^+^/K^+^-ATPase are involved. The interventional and dose-tapering studies in humans are necessary to elucidate the beneficial effects and mechanisms of selected cardiac steroids on human hearts.

## 6 Conclusion

Because of the specific modulation of Na^+^/K^+^-ATPase activity, Na^+^/K^+^-ATPase is a very intriguing drug target. The site of Na^+^/K^+^-ATPase modulation could be either Na^+^/K^+^-ATPase itself or downstream cascade pathways. The identification of pNaKtide as an antagonist of Na^+^/K^+^-ATPase signalling was the first step in this direction (123). Further cardiovascular damage could be avoided by inhibiting the Na^+^/K^+^-ATPase-Src oxidant amplification cascade (173). Additionally, it is important to assess the activity and expression of Na^+^/K^+^-ATPase and post-receptor cascades in distinctive specific and conjoint diseases, such as CMD, and further, evaluate the effects of different associated molecular targets’ inhibition or stimulation in such patients. The relationship between endogenous and exogenous CTS must be thoroughly investigated. Despite the restricted use of glycosides according to current guidelines recommended by specific cardiology associations, detecting some new CTS or elucidating some unknown effects recognizes the CTS as the focus of translational medicine trials.

## Author contributions

MO designed and wrote the paper, ESM, KB, and ZG wrote the paper, MR critically revised the paper, and EI designed and critically revised the paper. All authors contributed to the article and approved the submitted version.
